# Evaluation of a Boron-Conjugated SRC Inhibitor Combined with Proton and X-Ray Irradiation in U-87 MG and U-87 MG IDH1^R132H^ Glioma Cell Lines

**DOI:** 10.3390/ph19030392

**Published:** 2026-02-28

**Authors:** Cristiana Alberghina, Filippo Torrisi, Samuel Valable, Elsa Sarrazin, Isis Blanchard, Anthony Vela, Valentina Bravatà, Lorenzo Botta, Luca Lanzanò, Silvia Scalisi, Maria P. Demichelis, Maria G. Sabini, Iolanda V. Patti, Giorgio Russo, Francesco P. Cammarata, Rosalba Parenti

**Affiliations:** 1 Institute of Bioimaging and Complex Biological Systems, National Research Council, 90015 Cefalù, Italy; cristianaalberghina@cnr.it (C.A.); valentina.bravata@cnr.it (V.B.); giorgio-russo@cnr.it (G.R.); francescopaolo.cammarata@cnr.it (F.P.C.); 2 National Institute for Nuclear Physics (INFN), Laboratori Nazionali del Sud, 95123 Catania, Italy; mariagabriella.sabini@aoec.it (M.G.S.); iolanda.patti@aoec.it (I.V.P.); 3 Department of Pharmaceutical and Health Sciences, University of Catania, 95123 Catania, Italy; 4 ISTCT UMR6030, CYCERON, University of Caen Normandy, CNRS, Normandy University, 14000 Caen, France; sarrazin@cyceron.fr (E.S.); isis.blanchard@cnrs.fr (I.B.); 5 Department of Medical Physics, Centre François Baclesse, 14000 Caen, France; a.vela@baclesse.unicancer.fr; 6 Department of Ecological and Biological Sciences, University of Tuscia, 01100 Viterbo, Italy; lorenzo.botta@unitus.it; 7 Department of Physics and Astronomy «Ettore Majorana», University of Catania, 95123 Catania, Italy; luca.lanzano@dfa.unict.it (L.L.); silvia.scalisi@unict.it (S.S.); 8 Department of Physics «Alessandro Volta», University of Pavia, Via A. Bassi 6, 27100 Pavia, Italy; mariapaola.demichelis01@universitadipavia.it; 9 Medical Physics Unit, Cannizzaro Hospital, 95126 Catania, Italy; 10 Department of Biomedical and Biotechnological Sciences, University of Catania, 95123 Catania, Italy; parenti@unict.it

**Keywords:** glioblastoma, radiosensitivity, targeted therapy, DNA damage

## Abstract

**Background**: Adult diffuse gliomas represent one of the most aggressive types of brain tumors. Proton therapy offers a minimally invasive treatment option whose biological effectiveness may be enhanced through nuclear reactions involving boron atoms, leading to the emission of high-LET α-particles. In this study, we investigated the potential enhancement of radiation-induced damage of a novel boron-conjugated, ATP-competitive SRC kinase inhibitor, in the U-87 MG glioma cell line and its isogenic cell line stably expressing the IDH1 R132H mutation. **Methods**: Glioma cells were exposed to either proton or X-ray irradiation to assess whether any enhancement associated with this boron-delivery strategy was specific to proton interactions. Cell survival assays and analyses of DNA damage responses were conducted in both cell lines. **Results:** While no significant synergistic effects were observed in survival endpoints, differences emerged at the level of early DNA damage effects, with IDH1-mutant glioma cells displaying an enhanced acute response following combined treatment with proton irradiation. **Conclusions:** These findings support further pharmacological development of boron-based SRC-targeted strategies and underscore the importance of tailoring therapeutic approaches to specific glioma molecular subtypes.

## 1. Introduction

Glioblastoma (GBM) is the most aggressive malignant brain tumor among adult diffuse gliomas [1]. Despite advances in the molecular characterization of GBM and a better understanding of its pathogenesis, prognosis remains poor, with a median overall survival of only 14.6–20 months [2]. The current standard of care continues to follow the Stupp protocol since its publication in 2005, which involves maximal safe surgical resection followed by radiotherapy (RT) with concomitant temozolomide (TMZ) and subsequent adjuvant TMZ [3].

However, standard treatment modalities are not totally curative, and the resistance to both chemotherapy and RT plans is the main cause of GBM care failures that also depend on genetic and epigenetic backgrounds. Specifically, according to World Health Organization (WHO) guidelines, isocitrate dehydrogenase 1 and 2 (IDH1/2) mutation gene status distinguishes GBM IDH-wild type from astrocytoma or oligodendroglioma, which are IDH-mutant. The IDH1 gene mutation Arg132His is the most common gene alteration in gliomas. It is correlated with epigenetic modifications, assuming a prognostic value, along with other biomarkers such as MGMT, LOH 10q, epidermal growth factor receptor amplification, p16INK4a deletion, TP53 mutation, PTEN mutation, and the codeletion of 1p/19q [4,5].

A major challenge in GBM management lies in its highly infiltrative nature, which hampers complete surgical removal and complicates the delivery of local therapies while limiting off-target effects. This is particularly relevant for external-beam ionizing radiation in RT treatment. Moreover, charged particle therapies, such as protons, are among the precision modalities of RT, since they offer a more localized dose distribution [6]. However, the low relative biological effectiveness (RBE) of protons has been motivating research into synergistic strategies to enhance their therapeutic efficacy [7]. One such strategy is proton boron capture therapy (PBCT), based on the nuclear reaction between protons and the ^11^B isotope, producing three high-energy α-particles that can increase local cytotoxicity [8]. For this reason, PBCT represents an innovative treatment strategy designed to achieve physical-driven radiosensitization through the use of protons. It utilizes a nuclear fusion reaction involving low-energy protons and ^11^B atoms, thereby increasing the biological effectiveness of protons by releasing α-particles, which are expected to create DNA double-strand breaks. After initial in vitro studies validating PBCT effects [9], in vivo experiments reported encouraging data [10]. However, the selective delivery of boron atoms in the tumor area is the key critical challenge of its application, previously overcome by using boronated amino acids such as boronophenylalanine (BPA) [11].

In addition to the PBCT approach, our previous work on synergistic therapies focused on targeting the SRC protein tyrosine kinase in GBM cell lines using the Si306 molecule, a compound with specificity and favorable pharmacological properties [12,13]. Precisely, Si306 was able to inhibit SRC activity and enhance the effects of both X-ray and proton therapy against GBM cells [14,15], while maintaining activity against drug-resistant GBM cells [16,17]. Building on these findings, a boron-enriched SRC inhibitor, hereafter referred to as B-SRCi, has been developed by incorporating a cluster of ten boron atoms into the Si306 structure [18]. This dual-action compound is designed to inhibit SRC signaling while simultaneously enabling PBCT upon proton irradiation, thereby amplifying cytotoxicity. Preliminary experiments comparing boron-free Si306 with B-SRCi in U-251 MG cells showed promising results in clonogenic survival, but other biological effects were not explored [18]. In this study, we aimed to evaluate the therapeutic potential of the B-SRCi molecule in the PBCT setting. Specifically, we sought to confirm a potential enhancement of radiation effects between B-SRCi and protons by assessing radiation-induced DNA damage through the evaluation of γH2A.X foci formation. In addition, we decided to evaluate the differential therapeutic responses of B-SRCi and protons by comparing two GBM cell lines with different radiosensitivities. Indeed, we compared the human U-87 MG GBM cell line with an isogenic variant stably expressing the IDH1 R132H mutation. Thus, parallel experiments were performed with protons and X-rays to assess whether any synergistic effect was related to heavy-particle generation. In the first phase, we examined the impact of B-SRCi alone on cell growth in both lines to validate the previously established effective dose of 10 μM on U-251 MG [18]. We also performed gene expression assays for the SRC protein and its downstream target genes associated with cell death and proliferation processes. Secondly, we evaluated cell growth and survival rates of the two glioma cell lines, following treatment with protons and/or X-rays in combination with B-SRCi. Finally, we analyzed DNA damage induction and repair kinetics by quantifying γH2A.X foci in early (30 min) and late (2 h) time windows after irradiation treatments. In summary, our data indicate that B-SRCi is associated with an increased induction of early DNA damage in U-87 MG IDH1^R132H^ cells under proton irradiation, as reflected by enhanced γH2AX foci formation at early time points. While these findings suggest a possible contribution of boron-mediated proton interactions, the present study does not allow for a formal assessment of RBE or direct confirmation of the ^11^B(p,α)2α reaction in this experimental setting. Nevertheless, the observed context-dependent enhancement in IDH-mutant cells highlights the potential relevance of combining boron-conjugated SRC-targeting strategies with proton irradiation. In the framework of treatment personalization, further investigation of B-SRCi is warranted to better define its mechanistic contribution and to evaluate its potential dual mode of action in IDH-mutant gliomas.

## 2. Results

### 2.1. IDH1 and IDH1^R312H^ Expression on U-87 MG and U-87 MG IDH1^R312H^ Glioma Cell Lines

**Figure 1:**
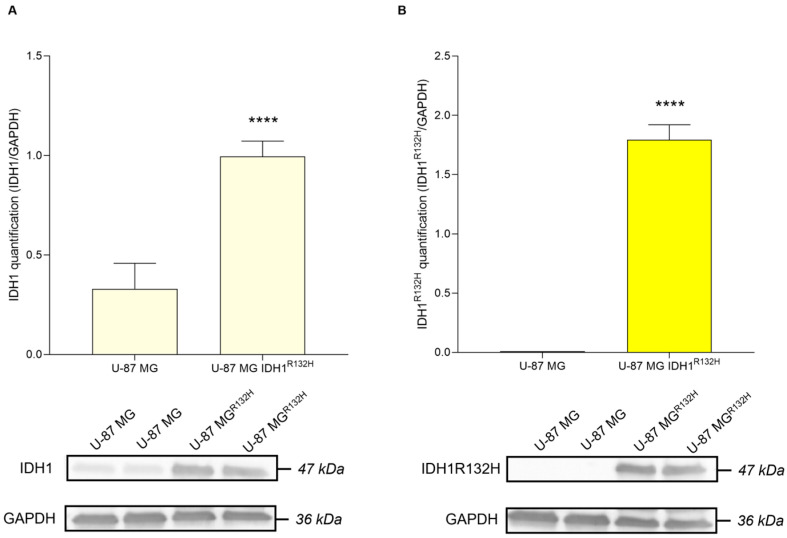
IDH1 and IDH1^R132H^ expression in U-87 MG and U-87 MG IDH1^R132H^. Quantification of IDH1 (**A**) and IDH1^R132H^ (**B**) normalized to GAPDH. Data are shown via interleaved bars plotting mean ± SD of *n* = 3 independent experiments; **** *p*-value < 0.0001 vs. IDH1.

First and foremost, to ensure that the IDH1^R132H^ transfection was successfully established and maintained in U-87 MG IDH1^R132H^, we performed a Western blot analysis. We confirmed that the expression of IDH1^R132H^ mutant protein was exclusively restricted to the U-87 MG IDH1^R132H^ cell line (Figure 1A,B). Densitometric quantification of the protein bands revealed a statistically significant increase in the expression of the IDH1^R132H^ mutant protein compared to wild-type IDH1 levels in both the U-87 MG and U-87 MG IDH1^R132H^ cells.

### 2.2. Basal Viability of U-87 MG and U-87 MG IDH1^R132H^ Cells Treated with B-SRCi

**Figure 2:**
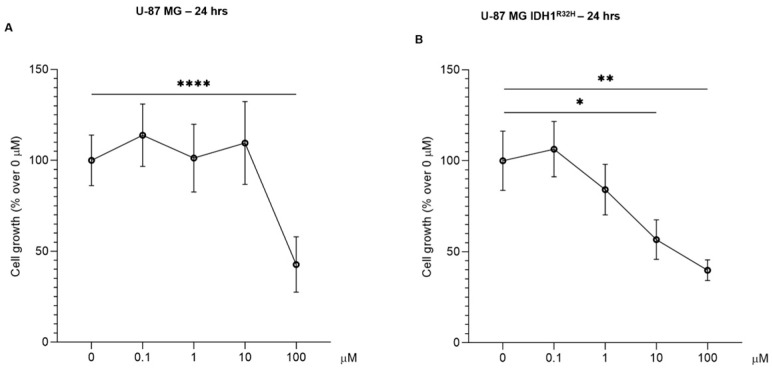
Cell growth evaluation after B-SRCi exposure. Evaluation of cell growth 24 h after B-SRCi treatment exposure at 0.1 µM, 1 µM, 10 µM, and 100 µM of (**A**) in U-87 MG and (**B**) U-87 MG IDH1^R132H^ lines; data were shown via column mean, error bars and mean connected ± SD of *n* = 3 independent experiments; * *p*-value < 0.05; ** *p*-value < 0.01; **** *p*-value < 0.0001 vs. 0 μM.

The B-SRCi compound was initially tested on the two cell lines, U-87 MG and U-87 MG IDH1^R132H^, to evaluate its effects on cell growth at increasing concentrations (0.1 µM, 1 µM, 10 µM and 100 µM). Cell growth was assessed using the trypan blue exclusion assay and cell counting after 24 h of exposure to B-SRCi (Figure 2). The results indicate that the U-87 MG cell line does not exhibit a reduction in cell growth after 24 h of treatment with B-SRCi at 0.1 µM, 1 µM, or 10 µM. Only the highest concentration, 100 µM, led to a marked and statistically significant decrease in cell growth compared to the control (Figure 2A). Conversely, the U-87 MG IDH1^R132H^ cell line showed greater sensitivity to the compound after 24 h of exposure. In fact, a statistically significant reduction in cell growth was observed at both 10 μM and 100 µM compared to the control (Figure 2B). While treatment with 100 µM B-SRCi markedly reduced cell growth in both cell lines, exposure to 10 µM preserved cell viability above 50% and caused detectable growth inhibition in U-87 MG cells. Accordingly, 10 µM was selected as the optimal concentration for subsequent B-SRCi experiments.

### 2.3. Boron Uptake Evaluation After B-SRCi Exposure

**Figure 3:**
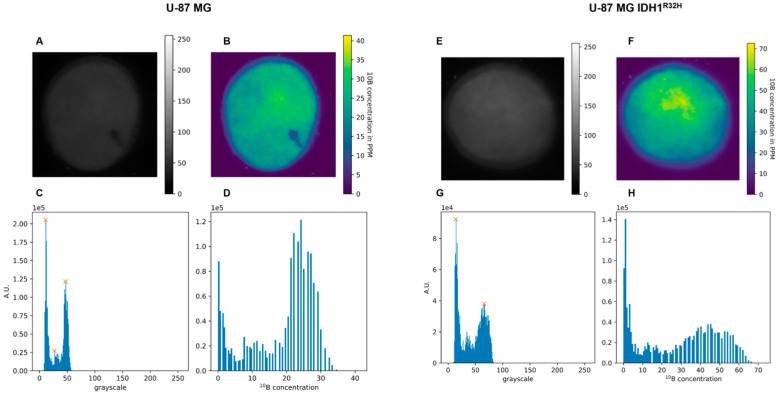
Boron uptake measurement in U-87 MG and U-87 MG IDH1^R132H^ cell lines. Grayscale image of the sample (intensity 0–255) (**A**,**E**); 2D map of the ^10^B concentration reconstructed using the calibration curve, expressed in ppm (color bar on the right) (**B**,**F**); histogram of the grayscale-level distribution (A.U.) within the analyzed area (the orange crosses indicate the main maxima (peaks) of the distribution, subsequently used to estimate the ^10^B concentration) (**C**,**G**); histogram of the distribution of ^10^B concentration values (ppm) corresponding to the pixels of the 2D map (**D**,**H**). The crosses mark the local maxima identified in the grayscale histogram. Each marked peak is used as a seed for a Gaussian fit performed in a neighborhood around the maximum, which provides a robust estimate of the peak position and amplitude for subsequent conversion to boron concentration.

The evaluation of boron uptake was performed to verify that the B-SRCi compound could serve as an effective boron delivery vehicle, a prerequisite for enabling proton–boron interactions relevant to PBCT and for interpreting the biological effects observed in subsequent experiments. Boron uptake was assessed in both the U-87 MG and U-87 MG IDH1^R132H^ cell lines following 24 h exposure to 10 µM B-SRCi, alongside untreated controls. No detectable signal was observed in the untreated samples, whereas reconstructed autoradiographic maps from treated cell pellets revealed a homogeneous signal distribution, consistent with uniform boron uptake across both cell lines (Figure 3).

Specifically, the quantification method allowed for the direct measurement of B^10^ and the indirect calculation of B^11^ content based on the known natural isotopic ratio of boron (20% is Boron^10^, and 80% is Boron^11^). In detail, the data in Table 1 show the numerical quantifications obtained in both cell lines treated with vehicle or 10 μM B-SRCi.

### 2.4. Evaluation of SRC Activation, Proliferation, and Apoptosis After B-SRCi Exposure

To evaluate the effects of SRC inhibition by B-SRCi on both cell lines, we evaluated the activation of pSRC and the expression of specific genes to evaluate the cell death effect and proliferation.

**Figure 4:**
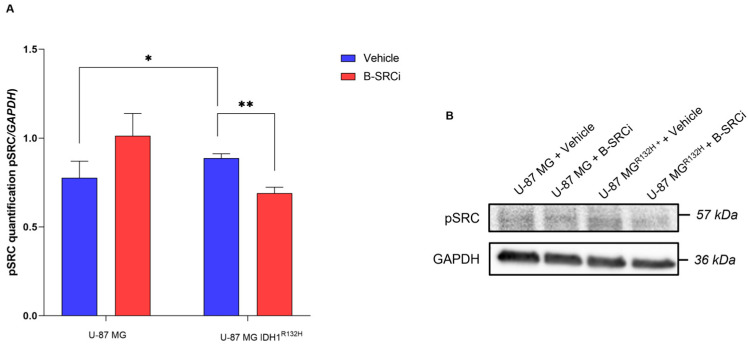
pSRC (Tyr416) expression in U-87 MG and U-87 MG IDH1^R132H^. (**A**) Quantification of pSRC protein normalized to GAPDH and (**B**) representative blots. Data are shown as interleaved bars plotting mean ± SD of *n* = 3 independent experiments; * *p*-value < 0.05 and ** *p*-value < 0.01.

SRC activation was assessed by measuring phosphorylation at Tyr416 (Figure 4). B-SRCi treatment resulted in differential modulation of SRC between the two cell lines. In U-87 MG cells, B-SRCi treatment did not significantly alter pSRC levels compared with vehicle-treated cells. In contrast, in U-87 MG IDH1^R132H^, B-SRCi significantly reduced pSRC levels relative to vehicle-treated cells. Additionally, baseline pSRC levels were significantly higher in U-87 MG IDH1^R132H^ vehicle-treated cells compared with U-87 MG vehicle controls (Figure 4).

**Figure 5:**
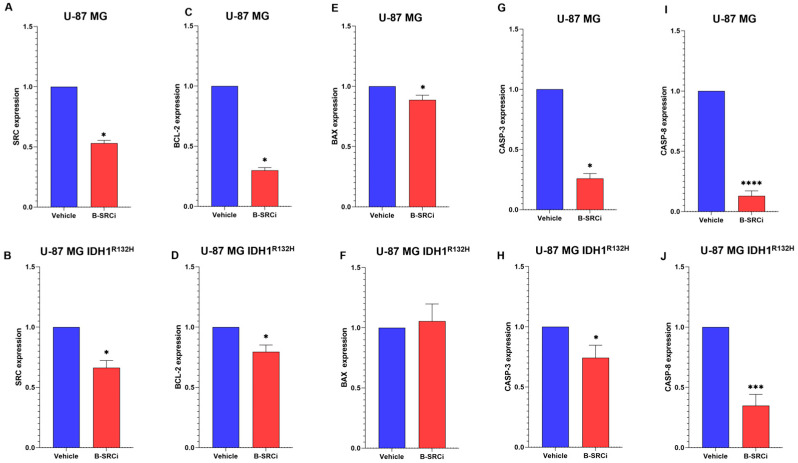
Gene expression evaluation after B-SRCi exposure. Evaluation of gene expressions in U-87 MG and in U-87 MG IDH1^R132H^ lines exposed to 10 µM B-SRCi treatment 24 h; evaluation of (**A**,**B**) SRC, (**C**,**D**) BCL-2, (**E**,**F**) BAX, (**G**,**H**) CASP-3, and (**I**,**J**) CASPASE-8 in both cell lines; data are shown as interleaved bars, mean ± SD of *n* = 3 independent experiments; * *p*-value < 0.05; *** *p*-value < 0.001; **** *p*-value < 0.0001 vs. vehicle (0 μM).

The results on both cell lines showed comparable effects for gene expression. In fact, both U-87 MG and U-87 IDH1^R321H^ cells showed a significant reduction in SRC (Figure 5A,B) and similar effects on genes driving proliferation and apoptosis processes. Both cell lines showed a statistically significant reduction in BCL-2, which can lead to a reduction in proliferation (Figure 5C,D). Regarding apoptotic effects after B-SRCi treatment, a statistically significant decrease in BAX or no significant change in U-87 and U-87 IDH1^R321H^ cells were observed, respectively (Figure 5E,F). This trend can be associated with a lack of activation of cell death mechanisms, as confirmed by the statistically significant reduction in CASPASE-3 (CASP-3) (Figure 5G,H) and CASPASE-8 (CASP-8) (Figure 5I,F) in both cell lines.

**Figure 6:**
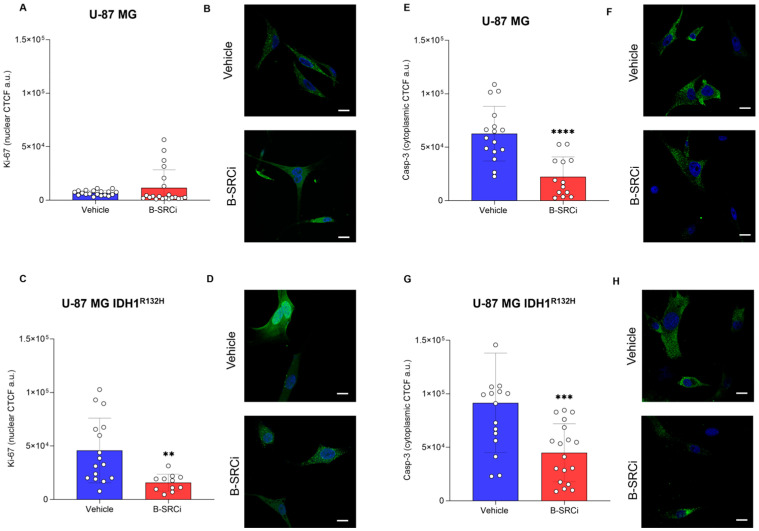
Immunofluorescence evaluation in U-87 MG and in U-87 MG IDH1^R132H^ lines exposed to 10 µM B-SRCi treatment 24 h; Ki-67 nuclear Corrected Total Cellular Fluorescence in Arbitrary Units (CTCF a.u.) of U-87 MG (**A**,**B**) and U-87 MG IDH1^R132H^ (**C**,**D**); CASP-3 cytoplasmic Corrected Total Cellular Fluorescence in Arbitrary Units (CTCF a.u.) of U-87 MG (**E**,**F**) and U-87 MG IDH1^R132H^ (**G**,**H**) data are shown as interleaved bars, mean ± SD of *n* = 3 independent experiments; ** *p*-value < 0.01; *** *p*-value < 0.001; **** *p*-value < 0.0001 vs. vehicle (0 μM). Nuclei are stained with DAPI (blue). Caspase and Ki67-positive cells are shown in green (Scale bar 20 μm).

We performed immunofluorescence assays for Ki-67 and CASP-3 to investigate the effects of B-SRCi on cell proliferation and cell death. Ki-67 analysis revealed a significant decrease in signal following B-SRCi treatment in U-87 IDH1^R321H^ (Figure 6A,B), whereas no significant change was observed in U-87 MG cells (Figure 6C,D). CASP-3 fluorescence intensity was significantly reduced in both cell lines (Figure 6E–H).

### 2.5. Cell Growth and Survival Evaluation of Proton and X-Ray Irradiation Combined with B-SRCi

**Figure 7:**
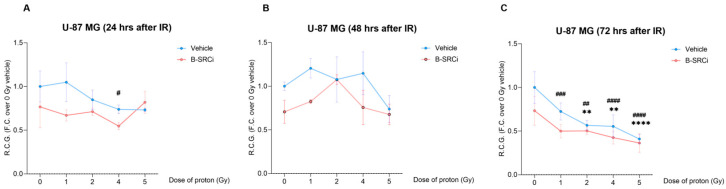
Rate of cell growth of proton-irradiated U-87MG cell line in combination with B-SRCi. Evaluation of R.C.G. in U-87 MG cell line exposed to 10 µM B-SRCi combined with protons at doses of 1, 2, 4, and 5 Gy after 24 h (**A**), 48 h (**B**), and 72 h (**C**); data are plotted as points and connecting lines, with error bars showing the mean ± SD of *n* = 3 independent experiments; ** *p*-value < 0.01 and **** *p*-value < 0.0001 for Vehicle + radiation dose vs. 0 Gy Vehicle (sham-irradiated); ^#^ *p*-value < 0.05, ^##^ *p*-value < 0.01, ^###^ *p*-value < 0.001 and ^####^ *p*-value < 0.0001 for B-SRCi + radiation dose vs. 0 Gy vehicle (sham-irradiated); R.C.G rate of cell growth.

To evaluate the effect of the compound and its potential synergistic interaction with protons, we analyzed cell growth by measuring the Rate Cell Growth (R.C.G.) at 24, 48, and 72 h. R.C.G in U-87 MG cells treated with protons alone or in combination with B-SRCi (Figure 7). At 24 h post-irradiation (Figure 7A), only a mild reduction in R.C.G. was observed, with a significant decrease detected for B-SRCi-treated cells at 4 Gy, compared to the control 0 Gy; at 48 h, both treatment groups showed no significant decline in R.C.G. across increasing proton doses, and differences between the vehicle and B-SRCi conditions were not statistically significant (Figure 7B). At 72 h, no significant reduction was observed for the vehicle-treated cells at 1 Gy, while reductions were observed for B-SRCi-treated cells at all doses, including 1 Gy. Furthermore, a more significant reduction was reported for the 4 Gy + B-SRCi group than for the 4 Gy proton-only group (Figure 7C).

**Figure 8:**
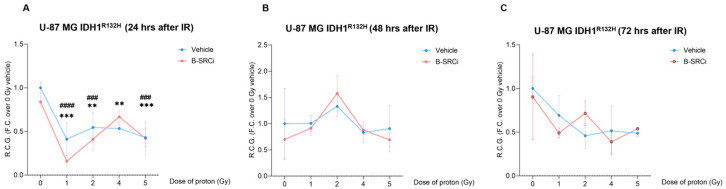
Rate of cell growth of the proton-irradiated U-87 MG IDH1^R132H^ cell line in combination with B-SRCi. Evaluation of R.C.G. in U-87 MG IDH1^R132H^ cell lines exposed to 10 µM B-SRCi combined with protons at doses of 1, 2, 4, and 5 Gy after 24 h (**A**), 48 h (**B**), and 72 h (**C**); data are plotted as points and connecting lines, with error bars showing the mean ± SD of *n* = 3 independent experiments; ** *p*-value < 0.01 and *** *p*-value < 0.001 for Vehicle + radiation dose vs. 0 Gy vehicle (sham-irradiated); ^###^ *p*-value < 0.001 and ^####^ *p*-value < 0.0001 for B-SRCi + radiation dose vs. 0 Gy vehicle (sham-irradiated); R.C.G rate of cell growth.

Proton treatments on U-87 MG IDH1^R132H^ cells, with and without the compound (B-SRCi), resulted in significant reductions in R.C.G. after 24 h at doses of 1, 2, 4, and 5 Gy (Figure 8A). Compared to 0 Gy, a greater reduction in R.C.G was reported in the combined treatment between B-SRCi and protons at doses of 1 Gy, 2 Gy, and 5 Gy (Figure 8A). However, no significant differences were observed between proton treatment alone and proton treatment combined with B-SRCi. Notably, the combined treatment did not result in a significant reduction in R.C.G. at the 4 Gy dose (Figure 8A). At 48 and 72 h, no significant decreases in R.C.G. were observed for any of the treatment conditions (Figure 8B,C).

**Figure 9:**
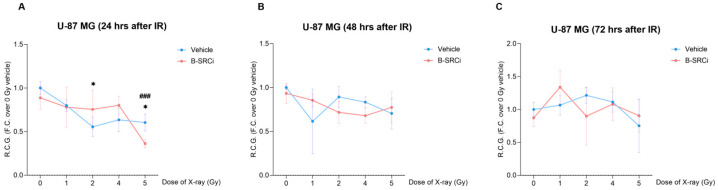
Rate of cell growth of the X-ray-irradiated U87-MG cell line in combination with B-SRCi. Evaluation of R.C.G. in U-87 MG cell lines exposed to 10 µM B-SRCi combined with X-ray at 1, 2, 4, and 5 Gy after 24 h (**A**), 48 h (**B**), and 72 h (**C**); data are plotted as points and connecting lines, with error bars showing the mean ± SD of *n* = 3 independent experiments; * *p*-value < 0.05 for Vehicle + radiation dose vs. 0 Gy vehicle (sham-irradiated); ^###^ *p*-value < 0.001 for B-SRCi + radiation dose vs. 0 Gy vehicle (sham-irradiated); R.C.G rate of cell growth.

A parallel analysis was conducted to evaluate the combined effects of X-ray irradiation and B-SRCi in both cell lines. In U-87 MG cells, X-ray treatment, both alone and in combination with B-SRCi, resulted in significant reductions in R.C.G. at 24 h following a 5 Gy dose and a higher significant reduction in 5 Gy + B-SRCi compared to the control at 0 Gy (Figure 9A). In contrast, a dose of 2 Gy significantly reduced R.C.G. only in the absence of the compound (Figure 9A). No significant reductions in R.C.G. were observed at 48 or 72 h post-treatment in these cells (Figure 9B,C).

**Figure 10:**
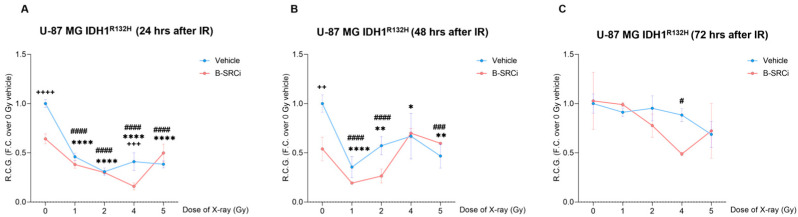
Rate of cell growth of the X-ray-irradiated U-87 MG IDH1^R132H^ cell line in combination with B-SRCi. Evaluation of R.C.G. in U-87 MG IDH1^R132H^ cell lines exposed to 10 µM B-SRCi combined with X-ray at 1, 2, 4, and 5 Gy after 24 h (**A**), 48 h (**B**), and 72 h (**C**); data are plotted as points and connecting lines, with error bars showing the mean ± SD of *n* = 3 independent experiments; ^++++^ *p*-value < 0.0001, ^+++^ *p*-value < 0.001 and ^++^ *p*-value < 0.01 for Vehicle vs. B-SRCi at the same dose; * *p*-value < 0.05, ** *p*-value < 0.01 and **** *p*-value < 0.0001 for Vehicle + radiation dose vs. 0 Gy vehicle (sham-irradiated); ^#^ *p*-value < 0.05, ^###^ *p*-value < 0.001 and ^####^ *p*-value < 0.0001 for B-SRCi + radiation dose vs. 0 Gy vehicle (sham-irradiated); R.C.G rate of cell growth.

In U-87 MG IDH1^R132H^, X-ray irradiation, both alone and combined with B-SRCi, resulted in significant reductions in R.C.G. at all tested doses (1, 2, 4, and 5 Gy) in U-87 MG IDH1^R132H^ after 24 h. Furthermore, a significant decrease in R.C.G. was observed when comparing 0 Gy to B-SRCi-treated cells. A further significant reduction was observed with 4 Gy X-ray irradiation combined with B-SRCi compared with 4 Gy X-ray alone (Figure 10A). At 48 h, a significant difference was found between sham-irradiated cells and B-SRCi-treated cells. Both alone and combined with B-SRCi, this treatment resulted in significant reductions in R.C.G. at all tested doses, except for the 4 Gy + B-SRCi group, which no longer showed a significant reduction (Figure 10B). At 72 h, a significant reduction in R.C.G. was observed only for the 4 Gy + B-SRCi treatment compared to the control (Figure 10C).

To further investigate the effects of the B-SRCi compound in combination with irradiation on cell viability, we performed a crystal violet survival assay. Proton irradiation significantly reduced the viability of U-87 MG cells at doses of 2, 4, and 5 Gy compared to the control (0 Gy). A significant reduction in viability was also observed at the same doses when combined with B-SRCi. A modest trend toward decreased viability in the combined treatment (B-SRCi + protons) compared with proton treatment alone was noted across all doses (Supplementary Figure S1A). In U-87 MG IDH1^R132H^ cells, significant reductions in viability were observed at 1, 2, and 5 Gy, with and without B-SRCi. However, the 4 Gy dose did not lead to a decrease in viability compared to the control. Furthermore, combining proton irradiation with B-SRCi did not result in a greater reduction in cell viability compared to irradiation alone (Supplementary Figure S1B). Survival analysis following X-ray irradiation did not reveal significant changes in either U-87 MG or U-87 MG IDH1^R132H^ cell lines (Supplementary Figure S2A,B). Specifically, only the 5 Gy dose led to a significant reduction in viability in U-87 MG cells, both in the group treated with irradiation alone and in the group that received the combined treatment with B-SRCi (Supplementary Figure S2A).

### 2.6. DNA Damage of Proton and X-Ray Irradiation Combined with the B-SRCi Compound

At this stage, after analyzing the effects of treatments on cell growth and survival, we moved on to evaluating DNA damage. To evaluate the kinetics of DNA damage and repair induced by treatments, we evaluated γH2AX foci formation using an early (30 min) and a later time point (2 h) after irradiation to analyze acute effects.

**Figure 11:**
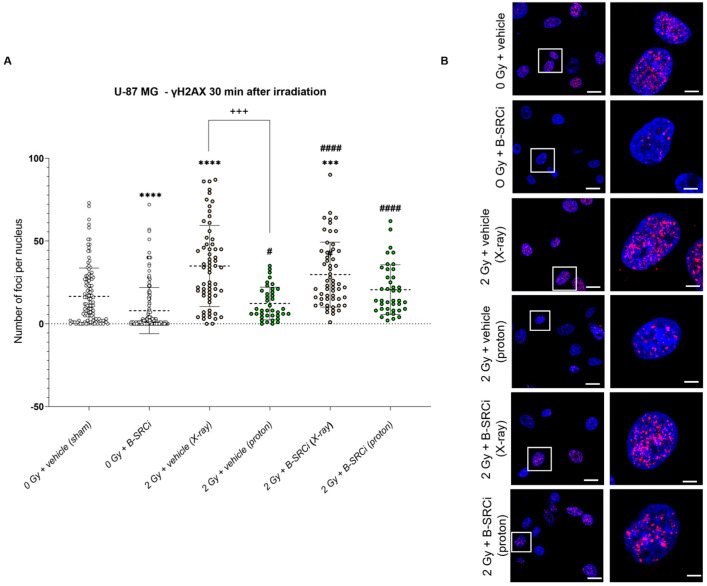
DNA damage by γH2A.X evaluation 30 min after X-ray and proton irradiation of U87-MG and the cell line in combination with B-SRCi. Evaluation of mean number of γH2A.X per nucleus in U-87 MG cell lines (**A**) and representative images (**B**) 30 min after irradiation with X-rays and protons at 2 Gy with and without 10 µM B-SRCi treatment; data are shown as a scatter plot, mean ± SD of *n* = 3 independent experiments; **** *p*-value < 0.0001 and *** *p*-value < 0.001, vs. 0 Gy vehicle (sham-irradiated); #### *p*-value < 0.0001 and # *p*-value < 0.05, vs. B-SRCi; +++ *p*-value < 0.001. Nuclei are stained with DAPI (blue). γH2AX foci are detected in red (anti-rabbit IgG–Atto 594). Scale bar 20 μm (5 μm for ROI).

At the early time point of 30 min post-irradiation, γH2AX foci analysis in U-87 MG cells showed a significant increase in DNA damage following X-ray irradiation, either alone or in combination with B-SRCi, compared with sham-irradiated controls. Proton irradiation alone and combined with B-SRCi induced a significantly higher number of foci compared with B-SRCi. Notably, X-ray irradiation induced a significantly higher number of foci compared with proton irradiation. However, no significant differences were observed between proton and X-ray treatments when combined with B-SRCi (Figure 11A,B). The analysis of γH2AX foci size showed trends consistent with those observed for foci number, indicating comparable modality-dependent effects at this early time point for U-87 MG (Supplementary Figure S3).

**Figure 12:**
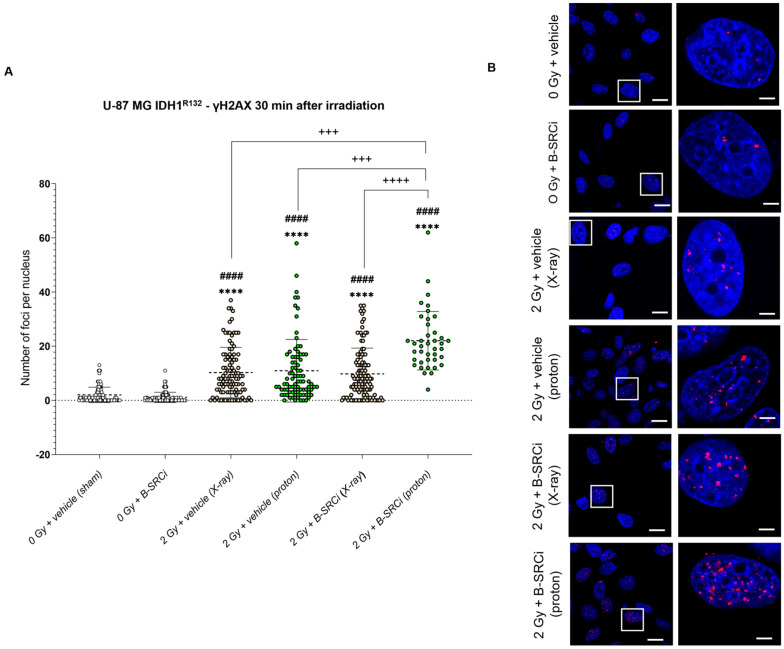
DNA damage by γH2A.X evaluation 30 min after X-ray and proton irradiation of U87-MG IDH1^R1321H^ cell line in combination with B-SRCi. Evaluation of mean number of γH2A.X per nucleus in U87-MG IDH1^R1321H^ cell lines (**A**) and representative images (**B**) 30 min after irradiation with X-rays and protons at 2 Gy with and without 10 µM B-SRCi treatment; data are shown as a scatter plot, mean ± SD of *n* = 3 independent experiments; **** *p*-value < 0.0001 vs. 0 Gy vehicle (sham-irradiated); #### *p*-value < 0.0001 vs. B-SRCi; +++ *p*-value < 0.001 and ++++ *p*-value < 0.0001. Nuclei are stained with DAPI (blue). γH2AX foci are detected in red (anti-rabbit IgG–Atto 594). Scale bar 20 μm (5 μm for ROI).

Analysis of γH2AX foci formation 30 min after irradiation in U-87 MG IDH1^R132H^ cells demonstrated that both proton and X-ray irradiation, either alone or in combination with B-SRCi, induced a significantly higher mean number of foci per nucleus compared with sham controls and B-SRCi-treated cells. Interestingly, proton irradiation combined with B-SRCi resulted in a statistically significant increase compared with proton irradiation and with X-ray irradiation with and without B-SRCi (Figure 12A,B).

Similarly, the pattern observed for the foci number was mirrored in the analysis of the mean foci size. Indeed, proton irradiation combined with B-SRCi resulted in a significantly larger mean foci size compared with all other experimental conditions (Supplementary Figure S4).

**Figure 13:**
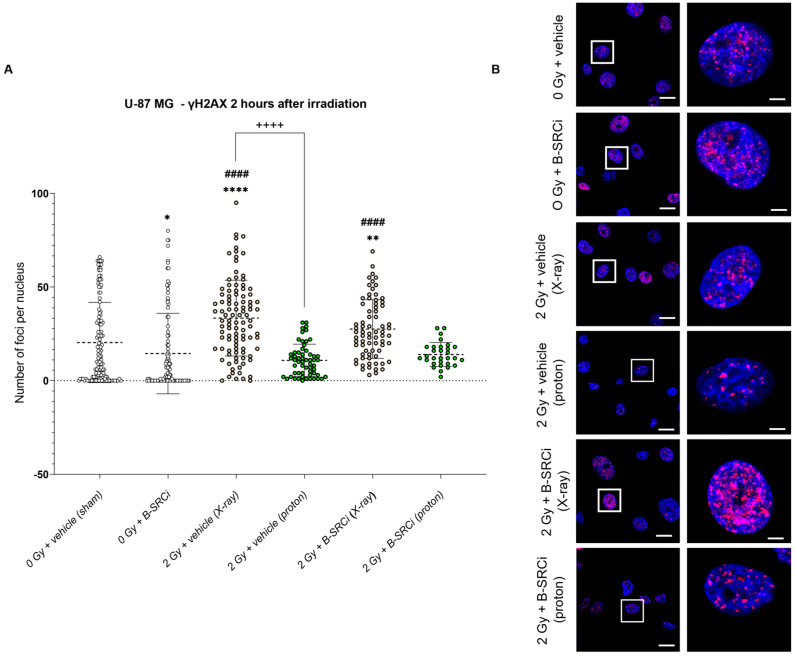
DNA damage by γH2A.X evaluation 2 h after X-ray and proton irradiation of the U87-MG cell line in combination with B-SRCi. Evaluation of the mean number of γH2A.X per nucleus in U87-MG cell lines (**A**) and representative images (**B**) 2 h after irradiation with X-rays and protons at 2 Gy with and without 10 µM B-SRCi treatment; data are shown as a scatter plot, mean ± SD of *n* = 3 independent experiments; * *p*-value < 0.05, ** *p*-value < 0.01 **** *p*-value < 0.0001 vs. 0 Gy vehicle (sham-irradiated); #### *p*-value < 0.0001 vs. B-SRCi; ++++ *p*-value < 0.0001. Nuclei are stained with DAPI (blue). γH2AX foci are detected in red (anti-rabbit IgG–Atto 594). Scale bar 20 μm (5 μm for ROI).

Analyses performed 2 h after irradiation in U-87 MG cells showed no statistically significant differences in the number of γH2AX foci per nucleus following proton irradiation. In contrast, X-ray irradiation, both alone and in combination with B-SRCi, resulted in a significant increase in γH2AX foci compared with sham-irradiated and B-SRCi–treated controls. Furthermore, treatment with 2 Gy X-rays induced a significantly higher number of foci than 2 Gy proton irradiation (Figure 13A,B). Regarding γH2AX foci size, the overall pattern was comparable to that observed for foci number. However, proton irradiation alone resulted in a significantly larger mean foci size compared with both sham- and B-SRCi–treated controls. No significant differences were observed in foci size between the irradiation-only treatment groups (Supplementary Figure S5).

**Figure 14:**
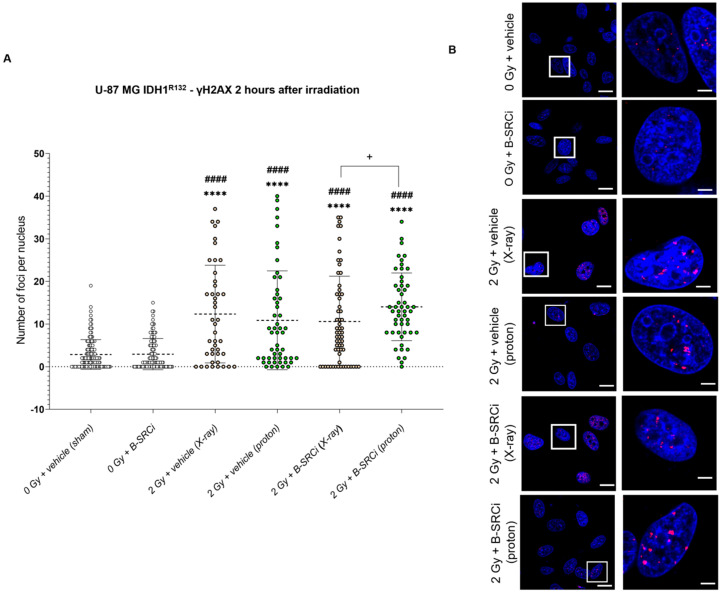
DNA damage by γH2A.X evaluation 2 h after X-ray and proton irradiation of the U87-MG IDH1^R1321H^ cell line in combination with B-SRCi. Evaluation of mean number of γH2A.X per nucleus in the U87-MG IDH1^R1321H^ cell lines (**A**) and representative images (**B**) 2 h after irradiation with X-rays and protons at 2 Gy with and without 10 µM B-SRCi treatment; data are shown as a scatter plot, mean ± SD of *n* = 3 independent experiments; **** *p*-value < 0.0001 vs. 0 Gy vehicle (sham-irradiated); #### *p*-value < 0.0001 vs. B-SRCi; + *p*-value < 0.05. Nuclei are stained with DAPI (blue). γH2AX foci are detected in red (anti-rabbit IgG–Atto 594). Scale bar 20 μm (5 μm for ROI).

In U-87 MG IDH1^R132H^ cells, the effect of the combined treatment with B-SRCi and protons was attenuated at 2 h post-irradiation compared with the response observed at 30 min. At this time point, all irradiated conditions exhibited a statistically significant increase in γH2AX foci number compared with control groups, but we did not observe a statistical difference between proton treatment alone versus proton treatment combined with B-SRCi. Notably, the combination of proton irradiation and B-SRCi resulted in a significantly higher number of foci than the combination of X-ray irradiation and B-SRCi (Figure 14A,B). The analysis of γH2AX foci size showed a pattern consistent with that observed for the foci number (Supplementary Figure S6).

## 3. Discussion

The depth–dose distribution of proton beams enables the precise irradiation of aggressive brain tumors such as gliomas with reduced exposure to healthy tissue, ultimately impacting patient survival and quality of life [19]. Moreover, when protons interact with boron atoms, they can generate ionizing radiation with substantially higher linear energy transfer (LET) through the production of α-particles, a mechanism that underlies proton boron capture therapy (PBCT) [20]. PBCT remains an emerging therapeutic modality, but early in vitro and in vivo preclinical studies have shown promising outcomes [10,21]. The development of effective therapeutic strategies for gliomas must also account for the distinct treatment responses associated with the IDH1 mutation, which represents a major stratification biomarker in the current classification of adult diffuse gliomas [22]. One of the most critical challenges in PBCT is achieving efficient and selective delivery of boron atoms into tumor tissue [23]. Previous studies have reported that the cell damage-enhancing effect of boron is highly variable and strongly dependent on cell type, metabolism, and intercellular signaling mechanisms [24]. In this context, we evaluated the therapeutic combination of protons and a boron-conjugated compound with a pyrazolo[3,4-d]pyrimidine ATP-competitive SRC kinase inhibitor (B-SRCi) in the U-87 MG glioma cell line (IDH1 wild type) and in its isogenic cell line stably expressing the IDH1 R132H mutation. B-SRCi has previously been tested in U-251 MG glioma cells, where clonogenic assays demonstrated a reduction in cell survival comparable to that obtained with the boron-free version of the inhibitor [18]. In the present study, we compared the effects of combining B-SRCi with either proton irradiation or X-rays in the two U-87 MG cell lines IDH1 wild type and IDH1^R132H^. Our aim was to determine if the potential synergistic effect of this boron-conjugated SRC inhibitor was specific to proton interactions, suggesting a PBCT-related effect, or whether similar results could be obtained with X-rays, implying a predominantly additive, but promising, effect of the scouting. We sought to evaluate whether these treatments had a diverse impact related to the IDH1 mutation status.

Firstly, as an initial step, we validated the expression of the IDH1^R132H^ mutation in the transfected U-87 MG cell line. In addition to a previous study, where the presence of the mutation was assessed by RT-PCR and used to demonstrate that IDH1^R132H^ expression increases X-ray radiosensitivity under hypoxic conditions [25], our work confirmed the mutation at the protein level via Western blot analysis. Following validation of our in vitro model, we assessed the effect of the compound on cell viability in both glioma cell lines. Exposure to doses ranging from concentrations of 1 to 100 μM reduced cell growth in both cell lines, with the most pronounced decrease observed at 100 μM. Based on these results and prior studies with the SRC inhibitor [15,26], we opted to select a dose of 10 μM for the irradiation-combination assays, as cell viability in both lines remained above 50% at this concentration after 24 h, to investigate the impact of B-SRCi on SRC signaling and downstream regulators of cell death and proliferation. The overall dose–response profile appeared predominantly cytostatic rather than cytotoxic, as no increase in apoptosis-associated markers was observed. These findings were further supported by immunofluorescence analysis, which demonstrated a reduction in Ki-67 and CASP-3 fluorescence signals in B-SRCi-treated U-87 IDH1^R132H^. In U-87 MG cells, a similar decrease in CASP-3 signal was observed, whereas Ki-67 levels remained unchanged. Previously, it was reported that the boron-free version of the drug at a dose of 10 μM did not cause significant increases in early or late apoptotic death 72 h after treatment and that the IC_50_ concentration was higher than 10 μM at 24 h of exposure [14,27]. Moreover, we assessed SRC activation by measuring phosphorylation at Tyr416 and performed RT-PCR analyses of SRC and selected downstream biomarkers. Our results indicate that B-SRCi modulates SRC activation in a cell line-dependent manner. Specifically, B-SRCi significantly reduced SRC Tyr416 phosphorylation in U-87 MG IDH1^R132H^ cells, whereas no significant reduction in SRC activation was observed in U-87 MG cells. Interestingly, despite the differential modulation at the phosphorylation level, treatment with 10 μM B-SRCi resulted in a statistically significant reduction in SRC mRNA expression in both cell lines compared with their respective controls. The observed downregulation of SRC mRNA may reflect transcriptional feedback mechanisms following pathway modulation rather than a direct transcriptional effect of the compound. Indeed, Src/SFK inhibitors such as PP2 and dasatinib have been reported to reduce Src expression both in vitro and in vivo [28,29]. Notably, although U-87 MG IDH1^R132H^ cells have been reported to exhibit increased dependency on SRC signaling for growth and survival [30], our study was not designed to comprehensively dissect SRC downstream signaling networks. A more comprehensive characterization of SRC-regulated signaling cascades and broader transcriptional profiling would be required to fully delineate context-dependent signaling differences between wild-type and IDH-mutant cells. Finally, when analyzing SRC signaling, it is essential to consider the impact of irradiation, as ionizing radiation has been shown to modulate SRC pathway activation. Previous studies have demonstrated that SRC inhibition in glial tumor cells reduces invasive capacity, in part by modulating the SRC–EGFR axis. In this context, the present evaluation of B-SRCi builds on these findings by exploring the potential interaction between SRC-targeting strategies and irradiation 16951163 [31]. Since the cell lines showed limited ability to perform clonogenic assays, we conducted a rate cell growth assay (R.C.G.). Specifically, these glioma cell lines, and particularly the IDH1-mutant subtype, are not prone to colony formation in standard conditions, displaying low attachment-independent growth and a IDH1 mutation-dependent compromise of morphology and proliferation [32]. In experiments combining treatment with protons, no significant differences were observed with B-SRCi co-treatment in either cell line. In turn, we observed a greater reduction in R.C.G. with co-treatment than with proton exposure alone in both cell lines. However, because these differences were not statistically significant relative to proton-only treatment, the effect appears additive rather than synergistic. Thus, these additive effects were less apparent following X-ray irradiation in U-87 MG cells but became more pronounced in U-87 MG IDH1^R132H^ cells, particularly at 24 h post-irradiation. These results suggested that the observed differences are more likely attributable to intrinsic variations in cellular radiosensitivity than to any synergistic action of B-SRCi, in line with our previous publication [25]. A similar result was observed in the investigation of survival using the crystal violet assay, where a trend toward greater reduction in viability was observed in the case of protons in combination with B-SRCi. The most encouraging finding emerged from the analysis of acute DNA-damage responses, assessed via γ-H2A.X foci formation at 30 min. When directly comparing proton and photon irradiation in the presence or absence of B-SRCi, our data indicate that, at the early time point of 30 min post-irradiation, U-87 MG IDH1^R132H^ cells treated with protons in combination with B-SRCi exhibited a significantly higher number of γH2AX foci compared with all other experimental conditions. This effect was not observed in U-87 MG cells, suggesting a context-dependent enhancement of early DNA damage induction in the mutant background. Our analyses were restricted to early DNA damage response endpoints and did not include dose–response experiments or clonogenic survival assays. Therefore, a formal determination of RBE cannot be derived from these data. Rather, the observed increase in γH2AX foci number in IDH1-mutant cells likely reflects a relative enhancement of early DNA damage signaling following proton irradiation in the presence of the boron-conjugated compound. Since this specific enhancement was not seen with X-rays, the damage can be attributed to the nuclear reaction generated after the reaction between protons and boron atoms. The amplification of DNA damage in the IDH1^R132H^ cell line suggests a specific vulnerability of these cells to B-SRCi-mediated PBCT. This may be linked to altered DNA repair mechanisms or to metabolic stress often associated with the IDH1^R132H^. Further investigations incorporating dose–response analyses and long-term survival endpoints will be necessary to determine whether this early increase in DNA damage translates into a sustained radiobiological advantage.

Specifically, subsequent research should aim to elucidate the molecular basis of the observed IDH1-specific vulnerability. Since IDH1 mutations are known to induce the accumulation of 2-hydroxyglutarate (2-HG), it is crucial to investigate whether the complex DNA lesions generated by the B-SRCi-mediated PBCT are rendered lethal by this specific defect in DNA repair machinery. The IDH1^R132H^ mutation consistently demonstrated an increased radiosensitivity in human malignant glioma cells under normoxia and severe hypoxia across a panel of cell lines [33]. The mechanisms underlying the increased radiosensitivity have been partially attributed to elevated oxidative stress associated with reduced glutathione depletion. This depletion results from a shortage of NADPH, which is diverted toward the production of 2-HG by the neomorphic, mutated IDH1 enzyme [34]. In addition to promoting oxidative stress, the IDH1 mutation may contribute to radiosensitivity by downregulating molecular pathways such as Wnt/β-catenin [35], while simultaneously activating others, including the AKT–mTOR signaling [34]. These alterations suggest that targeted therapies, such as SRC inhibitors capable of modulating signaling networks perturbed by the IDH1 mutation, could play a significant role in influencing the treatment response of glioma. Indeed, SRC activation promotes survival, growth, and cell cycle progression via the activation of AKT and through RAS/mitogen-activated protein kinase (MAPK), which determines the activated form of pERK [36]. Importantly, the boron-based free-version SRC inhibitor demonstrated greater potency than dasatinib, which is the most prominent candidate for SRC family kinase, in inhibiting FAK in GBM cells, whereas dasatinib failed to suppress ERK activity [16]. Future studies should further characterize SRC pathway modulation by integrating comprehensive analyses of downstream signaling markers and by including comparative assessments with the unconjugated parent compound and established SRC inhibitors such as dasatinib. The biological findings should be complemented by comprehensive dosimetry data, since it has been shown that, at clinically realistic boron concentrations, alpha particle-generating reactions occur at extremely low frequency [24]. Because only a very small fraction of cells can be directly affected by the emitted α-particles, purely physical mechanisms are insufficient to explain the observed increase in cell mortality. This enhancement is more plausibly associated with biological amplification mechanisms and intercellular signaling processes, such as radiation-induced bystander effects, that can propagate and amplify the initial damage originating from the few directly affected cells [37]. However, the few synergistic effects observed in this work may depend not only on the position of the proton beam but also on the low doses used. In fact, in a previous study on U-87 MG exposed to proton irradiation combined with boron in the form of sodium mercaptododecaborate (BSH), it was reported that significant enhancement of cell killing occurs only in the Bragg peak, while this effect was absent in the plateau, where boron does not alter proton-induced survival curves [24]. This spatial selectivity suggests that the observed effects may depend on the proton energy spectrum achieved in our irradiation setup immediately upstream of the Bragg peak. In this region, the estimated LET was 4.6 keV/µm, corresponding to proton energies of approximately 110–129 MeV. Further studies are required to determine whether this energy range overlaps with that required to trigger the p + ^11^B → 3α reaction. Moreover, in the study by Zahradníček et al., the authors explicitly distinguished the boron source with natural BSH (^nat^BSH), thereby specifying the relative proportions of ^10^B and ^11^B. The authors demonstrated that the addition of 40 ppm natural boron, corresponding to 32 ppm ^11^B and 8 ppm ^10^B in the form of ^nat^BSH, resulted in a statistically significant enhancement of proton-induced cell killing in U-87 MG cells selectively at the Bragg peak of a 190 MeV proton beam, whereas virtually no effect was observed in the plateau region or following photon irradiation [24]. In our study, boron uptake measurements demonstrated that B-SRCi delivered substantially higher boron concentrations than those used in the aforementioned work. Specifically, U-87 MG cells accumulated approximately 98 ppm of ^11^B, while U-87 MG IDH1^R132H^ cells reached even higher levels (≈176 ppm ^11^B). This may indicate that increased intracellular boron concentration alone is insufficient to induce a robust PBCT effect in the absence of appropriate proton energy spectra. It is also important to acknowledge that we did not investigate the subcellular localization of B-SRCi. Given that the high-LET α-particles produced in PBCT have a range of only a few microns, their proximity to the nuclear DNA is a critical factor in determining the severity of biological damage. Furthermore, while our results may suggest a contribution from the nuclear reaction, the decomposition of the individual effects, specifically distinguishing the pharmacological SRC inhibition from the physical boron-capture effect, remains to be fully elucidated. Future investigations to map intracellular boron distribution will involve essential follow-up steps to characterize the precise mechanism of action of this dual-mode agent.

Moreover, our findings can be contextualized by comparison with the study by Pang et al., who investigated proton–boron interactions in radiation-resistant SQ-20B cells and in radiation-sensitive MCF-7 cancer cells after exposure to BSH [38]. Although the study was not conducted in glioma cell lines, it is noteworthy that the most consistent and statistically robust differences were observed at the early stage of DNA damage responses (30 min and 2 h after irradiation). This convergence suggests that, even in the absence of measurable synergy in clonogenic or cell growth rate endpoints, boron-containing compounds may modulate the complexity of proton-induced DNA damage, particularly in genetically or metabolically altered backgrounds such as IDH1 mutant cells.

From a translational perspective, in vivo studies must also investigate the biodistribution of B-SRCi, particularly its capacity to cross the blood–brain barrier and selectively accumulate within the tumor microenvironment, which is a prerequisite for maximizing the proton–boron nuclear reaction, while sparing healthy brain tissue. Advancing targeted therapies along this direction will be essential to fully characterize and realize the therapeutic potential of this approach.

## 4. Materials and Methods

### 4.1. Cell Culture

U-87 MG human GBM cell lines were purchased from American Type Culture Collections (ATCC, Manassas, VA, USA). U-87 MG IDH1^R132H^ mutants were obtained using a cloning and transfection protocol as reported in [25]. Both the U-87 MG and U-87 MG IDH1^R132H^ cell lines were cultured and maintained in an exponentially growing culture condition, at 37 °C in a humidified atmosphere containing 21% O_2_ and 5% CO_2_, and were subcultured in 25 cm^2^ standard tissue culture flasks.

### 4.2. Synthesis of B-SRCi

All reactions were performed in flame-dried glassware under a nitrogen atmosphere. Reagents were obtained from commercial suppliers (Merck Srl, Milan, Italy) and used without further purification. The reagent o-carborane was purchased from Merck. The initial compound, called SI306, was prepared following a reported procedure [12].

TLC chromatography was performed on precoated aluminum silica gel SIL G/UV254 plates (Macherey-Nagel & Co., Düren, Germany). The detection occurred via fluorescence quenching or development in a ninhydrin solution (0.2 g of ninhydrin in 99.5 mL ethanol and 0.5 mL acetic acid), PdCl_2_ in HCl (25% in MeOH), and phosphomolybdic solution (10% in EtOH). Merck silica gel 60 was used for flash chromatography (23–400 mesh). 1H NMR spectra were measured on a Bruker Avance DRX400 (400 MHz/100 MHz) spectrometer (Bruker Corporation, Billerica, MA, USA). Chemical shifts for protons are reported in parts per million (ppm, δ scale) and internally referenced to the chloroform (CDCl3) signal at δ 7.28 ppm. 1H-NMR spectra are reported in the following order: multiplicity and number of protons; signals were characterized as s (singlet), d (doublet), dd (doublet of doublets), ddd (doublet of doublet of doublets), t (triplet), m (multiplet), or bs (broad signal). Mass spectra (MS) data were obtained using an Agilent 1100 LC/MSD VL system (G1946C) at a flow rate of 0.4 mL/min using a binary solvent system of 95:5 methyl alcohol/water. UV detection was monitored at 254 nm. Mass spectra were acquired in positive and negative mode scanning over the mass range. The general scheme for preparing the boron cluster is illustrated in Supplementary Figure S7.

For the synthesis of compound **1**, p-Toluenesulfonic acid (PTSA) monohydrate (0.144 mmol; 0.1 equation) is added to a solution of 3-bromo-1-propanol (1.44 mmol; 1 equation) and dihydropyran (DHP, 1.44 mmol; 1 equation) in dichloromethane (DCM, 5.5 mL). The reaction is left at room temperature under magnetic stirring for 2 h. Afterward, the reaction mixture is washed with a solution of 4M NaOH (3 × 3 mL), water (3 × 3 mL), and brine (3 × 3 mL). The organic phase is then dried with Na_2_SO_4_ and filtered. The product, a pale-yellow oil, was used for the subsequent steps without further purification (Y = 64%).

For the synthesis of compound **2**, to a solution of o-carborane (0.19 mmol; 1 equation) in distilled tetrahydrofuran (THF, 0.4 mL), *n*-butyllithium (*n*-BuLi, 0.38 mmol; 2 equation) is added at 0 °C [39]. The reaction mixture is kept at 0 °C for 30 min and at room temperature for one hour. It is then cooled again to 0 °C for two hours before adding a solution of compound **1** in distilled THF (0.2 mL). The reaction mixture is stirred magnetically under an argon atmosphere at room temperature for 24 h. Finally, the solution is diluted with diethyl ether (Et_2_O, 5 mL) and washed with a saturated solution of ammonium chloride (NH_4_Cl, 3 × 3 mL). The crude product obtained, a white solid, is purified on a chromatographic column under isocratic conditions using a mixture of petroleum ether (EP)/ethyl acetate (EtOAc) at a ratio of 98:2 as the eluent (Y = 36%). 1H NMR (400 MHz, CDCl3): δ ppm 4.62 (t, *J* = 2.8 Hz, 1H), 3.92–3.86 (m, 2H), 3.57–3.51 (m, 2H), 2.96 (m, 1H), 2.19–2.12 (m, 2H), 2.03–1.93 (m, 2H), 1.85–1.81 (m, 2H); 1.77–1.72 (m, 4H). MS (ESI) m/z calcd. for [C10H17B10O2]+ = 279.21; found = 279.43.

For the synthesis of compound **3**, compound **2** (0.0873 mmol; 1 equation) and PTSA (0.1746 mmol; 2 equation) are dissolved in ethanol (EtOH, 0.8 mL). The solution is kept at room temperature and magnetically stirred under an argon atmosphere for 12 h. It is then diluted in EtOH (4 mL) and washed with a solution of 4M NaOH (3 × 2 mL), water (3 × 2 mL), and brine (3 × 2 mL). The product, a white solid, was used for the subsequent steps without further purification (Y = 99%). The general scheme for the synthesis of B-SRCi is illustrated in Supplementary Figure S8.

For the definitive synthesis of boron-conjugated SRC inhibitor (hereafter B-SRCi), anhydrous sodium bicarbonate (NaHCO_3_, 1 mmol; 6 equations) is added to a solution of SI3061 (0.17 mmol; 1 equation) in distilled DCM (15 mL). After 5 min of stirring at room temperature, the solution is cooled to 0 °C, and triphosgene (0.17 mmol; 1 equation) is added. After 30 min, the reaction mixture is brought to room temperature and magnetically stirred under an argon atmosphere for 12 h until the spot corresponding to the initial substrates disappears in TLC. After that, a solution of compound **3** (0.17 mmol; 1 equiv.) in distilled DCM is transferred to the reaction mixture using a double needle. The reaction is stopped after 16 h. The mixture is filtered over celite, the solvent is evaporated under reduced pressure, and the crude product obtained is purified on a chromatographic column under isocratic conditions using a mixture of DCM/MeOH (98:2) as the eluent. The product obtained is a colorless oil (Y = 54%). ^1^H NMR (400 MHz, CDCl_3_) *δ* (ppm): 7.95 (s, 1H), 7.48 (d, *J* = 8 Hz, 1H), 7.42 (m, 3H), 7.29 (m, 4H), 7.15 (d, *J* = 8.4 Hz, 1H), 5.51 (t, *J* = 6 Hz, 1H), 4.94 (dd, *J* = 8.8, 14 Hz, 1H), 4.71 (dd, *J* = 6, 14 Hz, 1H), 4.35 (t, *J* = 5.2 Hz, 2H), 3.69 (t, *J* = 4.4 Hz, 4H), 3.01 (m, 2H), 2.99 (m, 1H), 2.49–2.46 (m, 4H), 2.37 (m, 2H), 2.21–2.16 (m, 2H), 2.07–2.01 (m, 2H). MS (ESI) *m/z* calcd. for [C_31_H_32_B_10_BrClN_6_O_3_S]^+^ = 792.20; found = 792.35.

### 4.3. Cell Treatment: Irradiation Setting and Drug Administration

B-SRCi was synthesized and provided by Lead Discovery Siena (Siena, Italy) as a stock powder and was dissolved in dimethylsulfoxide (DMSO) (Merk, Milan, Italy) as previously described [18]. The compound was stocked in DMSO and diluted to the final concentration with fresh medium (no more than 0.5% DMSO), where GBM cells were maintained for 24 h in each test. Control samples for all biological tests were supplemented with the same vehicle (DMSO).

X-ray irradiation was performed using the linear accelerator, Elekta Synergy (Elekta AB, Stockholm, Sweden), at the Radiotherapy Department of Cannizzaro Hospital, Catania, Italy, at a dose rate of 3 Gy/min with a 6 MV X-ray. GBM cell irradiation was carried out using dose values of 0 Gy (as a sham-irradiated group), 1 Gy, 2 Gy, 4 Gy, and 5 Gy. After irradiation, cells were replaced with fresh medium to remove the B-SRCi and maintained under growing culture conditions until the end of the experiment.

Proton irradiations were performed on an IBA ProteusONE system at the CYCLHAD Hadrontherapy Center (Caen, France) in pencil beam scanning mode. The system uses a compact superconducting synchrocyclotron to generate a proton beam with energies up to 226 MeV. The dose is delivered using pencil beam scanning (PBS), where magnetically steered, energy-modulated proton beams scan the target volume layer by layer for highly conformal treatment. A 98.4 MeV monolayer beam was delivered over a 20 × 24 cm^2^ field at a dose rate of about 2 Gy/min, using a vertically upward beam incidence with pencil beam scanning (PBS), where magnetically steered, energy-modulated proton beams scanned the target volume layer by layer for highly conformal treatment. The LET was 4.6 keV/um. To modulate the LET at the sample entrance position, varying thicknesses of RW3 water-equivalent plates were used upstream, and the samples were placed directly on top of the RW3 plates during irradiation. The number of monitor units (MU) was adjusted to deliver the prescribed dose for each irradiation condition. Reported doses are given as physical dose (Gy).

### 4.4. Western Blot

For Western blot analysis, cells were seeded in 6-well plates at a final density of 5 × 10^5^/well, incubated at 37 °C, and processed as previously described [40]. The next day, 10 μM SRC-Bi was added to cells and maintained for 24 h. Then, cells were collected to obtain a dry pellet. Proteins were extracted using RIPA Lysis Buffer (50 μL/sample; Cat#ab156034, Abcam) supplemented with protease inhibitor (1:100, Cat#P8340, Merck, Darmstadt, Germany). Samples were incubated for 20 min at room temperature and centrifuged at 13,000× *g* for 3 min. A total of 15 μg of proteins were electrophoresed on 4–15% Mini-PROTEAN TGX gels (Cat#4561083, Bio-Rad, Hercules, CA, USA) and transferred to 0.2 μm nitrocellulose membranes of Trans-Blot Turbo Transfer Pack (Cat#1704158, Bio-Rad), using Trans-Blot Turbo Transfer System (Bio-Rad). Membranes were incubated for 1 h at room temperature with blocking buffer (5% non-fat milk in 0.1% tween-20 in PBS) and then overnight at 4 °C with primary antibodies diluted in blocking buffer. The primary antibodies, rabbit anti-IDH (Abcam, Cat# ab172964, RRID: AB_2864315, Cambridge, UK), mouse anti-IDH^R132H^ (Dianova, Cat. #DIA-H09, RRID: AB_2335716, 1:1000, Hamburg, Germany), rabbit anti-Phospho-Src (Tyr416) (Cell Signaling Technology, Cat.# 2101, RRID: AB_331697, 1: 1000, Danvers, MA, USA), and GAPDH (Abcam Cat.# ab181602, RRID: AB_2630358, 1:10,000), were used for wWestern blot. The next day, membranes were washed 3 times in 0.1% tween-20 in PBS and then incubated for 1 h at room temperature with the corresponding secondary antibody: goat anti-mouse IgG (H + L) secondary antibody, HRP (Cat.# 31430, Invitrogen, RRID: AB_228307, 1:5000) and anti-rabbit monoclonal antibody HRP (Cat.# 31460, Invitrogen, RRID: AB_228341, 1:10,000, Waltham, MA, USA). Protein bands were visualized using a ChemiDoc System (Bio-Rad), and protein levels were quantified via densitometric analysis. Fiji (version 2.14.0/1.54j) analysis software was used to quantify the density of each band, which was then normalized to the GAPDH optical density measured in the same membrane. All values are shown as the mean fold change (FC) over control ± SD.

### 4.5. Trypan Blue Exclusion Cell Growth

To evaluate the basal cytotoxicity effects of B-SRCi, cell numbers and viability were evaluated using the trypan blue exclusion assay. U-87 MG and U-87 MG IDH1^R132H^ cells were incubated at increasing compound concentrations of 0.1, 1.0, 10, and 100 μM for 24 h under growing culture conditions. The cells were plated in 6-well plates at a density of 50,000/cm^2^. The following day, the treatment was performed at the previously listed doses, and the plates were incubated for 24 h. Subsequently, the cells were counted using a Burker chamber, excluding cells stained with 0.4% trypan blue (T8154, Merck/Sigma-Aldrich, Darmstadt, Germany) at a 1:10 dilution.

### 4.6. Quantification of Boron Uptake

For boron uptake quantification, both the U-87 MG and U-87 MG IDH1^R132H^ cell lines were incubated in culture medium supplemented with 10 μM B-SRCi for 24 h. After incubation, cells were washed three times with PBS to remove extracellular boron. Cells were then detached, collected, and centrifuged to obtain compact pellets of 4 × 10^6^ cells. Pellets were deposited onto circular Mylar disks and allowed to dry overnight at room temperature. Vehicle treatments with DMSO were processed in parallel to verify the absence of boron contamination introduced during preparation and handling, thus ensuring the reliability of the measurements in treated samples.

Neutron autoradiography was performed using Solid State Nuclear Track Detectors (SSNTDs), passive detectors capable of recording the passage of charged particles as latent damage tracks. Upon irradiation with thermal neutrons, ^10^B present in the cell pellet undergoes the capture reaction ^10^B(n,α)^7^Li, generating charged particles that interact with the SSNTD and produce latent tracks in the detector material [41]. Dried pellets were placed in direct contact with the SSNTDs and irradiated in a thermal neutron field inside the thermal column of the TRIGA Mark II reactor (LENA laboratory, University of Pavia, Italy) [42]. The irradiation position was selected based on prior characterization of the thermal neutron field using experimental measurements and Monte Carlo calculations. For qualitative boron uptake mapping, samples were irradiated at a reactor power of 250 kW for 2 h in a position where the neutron flux is approximately 2 × 10^9^ cm^−2^ s^−1^ [43]. Irradiation was performed consistently across conditions to ensure comparability among samples and calibration standards.

After irradiation, detectors were chemically etched to reveal latent tracks. Etching was performed by immersing SSNTDs in NaOH solution at 70 °C for 20 min. Under these conditions, the etchant preferentially enlarges the damaged regions along particle tracks, making them observable by optical microscopy. Etching time and temperature were chosen to achieve sufficient sensitivity for qualitative mapping of boron distribution at the pellet scale. Following etching, detectors were rinsed thoroughly (e.g., with deionized water) to stop the reaction and remove residual NaOH, then dried prior to imaging.

Macroscopic autoradiography images of the full pellet area were acquired using a stereomicroscope (Leica, Wetzlar, Germany). Image areas typically ranged from 30 to 50 mm^2^, covering the pellet footprint and enabling the assessment of the spatial uniformity of boron distribution across the sample. Autoradiography images were analyzed as grayscale maps, where local grayscale intensity reflects local track density and, therefore, the relative boron content under fixed irradiation and etching conditions. For quantitative estimation of boron concentration, grayscale values were converted to boron concentration using a concurrent calibration performed with cell pellets of known boron concentration prepared and processed under identical conditions (Supplementary Figure S9). For visualization, boron distribution maps were displayed using the Viridis colormap (Matplotlib v3.10.3), with colors corresponding to predefined boron concentration ranges.

### 4.7. Gene Expression Analyses via Real-Time Quantitative Reverse Transcription Polymerase Chain Reaction (qRT-Polymerase Chain Reaction)

Twenty-four hours following each treatment, cells were collected and counted, and the pellet was promptly stored at −80 °C. Total RNA was isolated from the following experimental samples: U-87 MG and U-87 MG IDH1^R132H^ exposed to B-SRCi treatment, normalized with those not exposed, and assessed for its concentration and purity as previously described [44]. Total RNA was converted into cDNA using SuperScript II reverse transcriptase according to the manufacturer’s instructions and subsequently examined in triplicate utilizing a Fast 7500 Real-Time PCR System (Applied Biosystems, Carlsbad, CA, USA). Post-amplification melting-curve analysis was used to manage reaction specificity. The oligonucleotide primers selected for qRT-PCR were designed using Primer3 software (version 4.1.0) and checked for human specificity using the NCBI database and are available upon request. Quantitative data, normalized to the rRNA 18S housekeeping gene, were evaluated by averaging the triplicate cycle threshold (Ct) values using the 2^−ΔΔct^ method in SDS software (version 1.4, Applied Biosystems, Waltham, MA, USA). The information presented was obtained from three separate experiments, with values reported as the mean ± SD relative to mRNA levels in the untreated U-87 MG and U-87 MG IDH1^R132H^ used as control samples.

### 4.8. Rate of Cell Growth Assay

The rate of cell growth (R.C.G.) was evaluated by normalizing and expressing the treated samples as fold changes relative to the corresponding untreated (0 Gy) controls for each cell line and time point (24, 48, and 72 h). Briefly, U-87 MG and U-87 MG IDH1^R132H^ were seeded on six-multiwell plates at a final density of 2 × 10^5^ cells/well. Cells were seeded and counted considering three different time points: 24 h, 48 h, and 72 h. The R.C.G. was calculated by counting the number of viable cells by trypan blue vital stain exclusion and dividing it by the number of plated cells; ratios were divided by the number of days per passage [45].

### 4.9. Crystal Violet Assay

For the crystal violet assay, both U-87 MG and U-87 MG IDH1^R132H^ cell lines were plated at 60,000/well. Brief irradiation was performed the day after cell plating. Two days later, the medium was aspirated and washed with PBS; then, 400 µL of solution containing 2.3% crystal violet, 0.1% ammonium oxalate, and 20% ethyl alcohol (HT90132 Merck/Sigma-Aldrich) was added to stain and fix the cells for 25 min. Three washes with water were then performed to remove excess dye, and the plates were dried overnight at room temperature. The following day, the solution was solubilized by adding 400 µL of 10% acetic acid to the wells, and three 100 µL technical replicates were generated for each experimental point in a 96-well ELISA plate. After brief shaking, the wavelength was read at 590 nm with Spectro SPARK (Tecan, Männedorf, Switzerland)

### 4.10. Immunofluorescence

U-87 MG and U-87 MG IDH1^R132H^ cells were seeded onto sterile cover glasses in 24 multiwell plates at 100,000 cells/well. After 7 h, cells were treated with B-SRCi for 24 h. Cells were then irradiated with 2 Gy and fixed in paraformaldehyde 4% at 30 min and 2 h post-irradiation. Samples were then incubated with bovine serum albumin (BSA) 3%, Triton-X-100 0.5% in PBS as a blocking solution and to permeate cells for 1 h at room temperature. Immunostaining was performed using a primary antibody anti-H2A.X (Ser139) (phospho-H2A.X rabbit, polyclonal ab, PA5-77995-Invitrogen; 1:100), primary antibody anti-Caspase-3 (anti-Caspase-3 recombinant rabbit monoclonal antibody, 9H19L2-700182, Thermo Fisher, 1:250, Waltham, MA, USA), and primary antibody anti-Ki67 (anti-Ki67 rabbit monoclonal antibody [SP6], ab16667, Abcam; 1:500, Cambridge, UK) dissolved in BSA 1%, Tween 0.1% in PBS for 2 h at room temperature. Then, samples were washed three times with BSA 0.2% and Triton-X 100 0.05% in PBS for 10 min. Samples were incubated for 1 h with Anti-Rabbit IgG-Atto 594 (77671-1ML-F Sigma-Aldrich; 1:300) for phospho-H2A.X and with Goat anti-Rabbit IgG (H + L) Cross-Adsorbed Secondary Antibody, Alexa Fluor™ 488 (A-11008, Thermo Fisher) for Ki-67 and CASP-3. Nuclei were counterstained, adding 4’,6-diamidino-2-phenylindole (DAPI) stain (Thermo Fisher Scientific, 62248; 1:1000) for 15 min at room temperature. After two washes in PBS for 5 min, samples were mounted with ProLong Diamond Antifade Mountant (Invitrogen, P36965).

### 4.11. Confocal Analysis and Quantification of CASP-3, Ki67, and γH2A.X

Image acquisition was performed on a Leica TCS SP8 confocal laser scanning microscope (Leica Microsystems, Wetzlar, Germany). An HCX PL APO CS2 63 × 1.40 NA oil immersion objective (Leica Microsystems, Mannheim, Germany) was used. For γH2A.X–ATTO 594, the excitation wavelength was set at 561 nm, with an emission bandwidth of 589–643 nm. For DAPI, the excitation wavelength was set at 405 nm, with an emission bandwidth of 412–483 nm. Fluorescent emissions were detected with a photomultiplier (PMT) for the DAPI channel and a Hybrid Detector (HyD) for the ATTO 594 and 488 channels. The pinhole size was set to 0.8 Airy Units at a wavelength of 580 nm. Images were acquired with 2048 × 2048 pixels and a 45 nm pixel size.

The number of foci and the average foci size per nucleus were quantified using Fiji (version 2.14.0/1.54j). Analysis was performed using a custom macro script that enabled the selection of all nuclei within the defined regions of interest (ROIs). Within each ROI, individual γH2A.X foci were automatically detected and counted by the software. To minimize background noise and ensure consistency, identical color adjustments and image filters were applied across all samples. For CASP-3 and Ki-67, fluorescence intensity was quantified within ROIs using the Isodata thresholding algorithm in ImageJ software (version 1.54j). Cells were carefully segmented to separately measure nuclear and cytoplasmic fluorescence signals. Data were calculated by considering the Corrected Total Cellular Fluorescence (CTCF), expressed in Arbitrary Units (A.U.). CTCF was calculated as follows:

Integrated Density − (Area of selected cell × Mean fluorescence of background readings)

### 4.12. Statistical Analysis

Data analysis was performed using GraphPad Prism software version 8.2.1. Data were tested for normality using a Shapiro–Wilk normality test and subsequently assessed for homogeneity of variance. For data that failed the normality test, the Kruskal–Wallis test was used to compare groups. For comparison of *n* = 2 groups, the *t*-test was applied. For comparison of *n* > 2 groups, two-way analysis of variance (ANOVA) was used, followed by the Holm–Sidak post hoc test for multiple comparisons. Data are presented as the mean ± SD. A *p*-value of < 0.05 was considered statistically significant, and symbols used to indicate statistical differences are described in the figure legend.

## 5. Conclusions

Proton therapy leverages the inverted dose–depth profile of charged particles; however, its relatively low LET limits its radiobiological advantage over conventional photon therapy. Proton–boron capture therapy (PBCT), when combined with a molecularly targeted boron-conjugated compound, represents a promising strategy to locally increase LET while preserving the dose-sparing effect on surrounding healthy tissue. Our findings indicate predominantly additive interactions of B-SRCi with proton and X-ray irradiation in cell growth rate and viability assays in U-87 MG and U-87 MG IDH1^R132H^ cells. Although these additive responses varied between the two genetic backgrounds, reflecting differences in their intrinsic radiosensitivity, clear synergistic effects were generally not observed. The main differential effect emerged in the analysis of early DNA damage responses, where proton irradiation combined with B-SRCi resulted in a significant increase in the number and size of γH2AX foci in U-87 MG IDH1^R132H^ cells. This proton-specific enhancement was not observed with X-ray irradiation and was restricted to early time points, suggesting a context-dependent modulation of the acute DNA damage response. Importantly, given that the present study did not include dose–response analyses or long-term clonogenic survival assays, these findings should be interpreted as evidence of a comparative early biological effect rather than as a demonstration of enhanced relative biological effectiveness or durable therapeutic gain. In addition, while the present work focuses on the induction of acute DNA damage, future studies characterizing the impact of different radiation modalities on the SRC phosphorylation cascade, depending on the different IDH-status, should be conducted. This would be essential to fully evaluate the potential of B-SRCi to overcome radiation-induced signaling resistance. Overall, B-SRCi appears to modulate SRC signaling and early radiation-induced DNA damage responses in a cell line-dependent manner. Further studies incorporating extended repair kinetics, radiation dose–response analyses, and long-term survival endpoints will be required to determine whether the observed early effects translate into meaningful radiobiological advantages, particularly in IDH-mutant glioma models.

## Figures and Tables

**Table 1:** B^10^ and B^11^ quantification after exposure to 10 μM B-SRCi.

Cell Lines	B^10^ ppm (Mean ± SD)	B^11^ ppm (Mean ± SD)
U-87 MG + Vehicle	0 ± 0.1	0 ± 0.4
U-87 MG + B-SRCi	24.4 ± 0.3	98 ± 1
U-87 MG IDH1^R132H^ + Vehicle	0 ± 0.1	0 ± 0.4
U-87 MG IDH1^R132H^ + B-SRCi	44.1 ± 0.5	176 ± 2

## Data Availability

The original contributions presented in this study are included in the article and Supplementary Materials. Further inquiries can be directed to the corresponding authors.

## Supplementary Materials

The following supporting information can be downloaded at https://www.mdpi.com/article/10.3390/ph19030392/s1: Figure S1: Evaluation of cell survival in U-87 MG (A) and in U-87 MG IDH1R132H (B) cell lines irradiated with proton at 1, 2, 4 and 5 Gy after exposed to 10 μM of B-SRCi treatment; data were shown via interleaved bars, mean ± SD of *n* = 3 independent experiments; * *p*-value < 0.05; ** *p*-value < 0.01; *** *p*-value < 0.001, **** *p*-value < 0.0001 vs. 0 Gy vehicle (sham irradiated); Figure S2: Evaluation of cell survival in U-87 MG (A) and in U-87 MG IDH1R132H (B) cell lines irradiated with X-ray at 1, 2, 4 and 5 Gy after exposed to 10 μM of B-SRCi treatment; data were shown via interleaved bars, mean ± SD of *n* = 3 independent experiments; *** *p*-value < 0.001, vs. 0 Gy vehicle (sham irradiated); Figure S3: Average size of γH2A.X 30 min after proton and X-ray irradiation of U87-MG in combination B-SRCi; data were shown scatter plot, mean ± SD of *n* = 3 independent experiments; **** *p*-value < 0.0001, *** *p*-value < 0.001 and ** *p*-value < 0.01, vs. 0 Gy vehicle (sham irradiated); #### *p*-value < 0.0001 vs. B-SRCi; +++ *p*-value < 0.001; Figure S4: Average size of γH2A.X 30 minutes after proton and X-ray irradiation of U-87 MG IDH1R132H in combination B-SRCi; data were shown scatter plot, mean ± SD of *n* = 3 independent experiments; **** *p*-value < 0.0001 vs. 0 Gy vehicle (sham irradiated); #### *p*-value < 0.0001 vs. B-SRCi; + *p*-value < 0.05 and +++ *p*-value < 0.001; Figure S5: Average size of γH2A.X 2 hours after proton and X-ray irradiation of U-87 MG in combination B-SRCi.; data were shown scatter plot, mean ± SD of *n* = 3 independent experiments * *p*-value < 0.05, *** *p*-value < 0.001 **** *p*-value < 0.0001 vs. 0 Gy vehicle (sham irradiated); #### *p*-value < 0.0001 vs. B-SRCi; Figure S6: Average size of γH2A.X 2 h after X-ray and proton irradiation of U-87 MG IDH1R132H in combination B-SRCi; data were shown scatter plot, mean ± SD of *n* = 3 independent experiments; **** *p*-value < 0.0001 vs. 0 Gy vehicle (sham irradiated); #### *p*-value < 0.0001 vs. B-SRCi; Figure S7: Synthesis of the alcohol linker functionalized with o-carborane. Protection of the alcohol function of 3-bromo-1-propanol with DHP (A) is followed by nucleophilic attack of the boron cluster and displacement of the bromide atom (B). Finally, the functionalized linker is obtained by removing the protective group, restoring the hydroxyl moiety (C); Figure S8: Detailed representation of the synthesis of an SI306 derivative hybridized with a boron cluster via the triphosgene route to give B-SRCi; Figure S9. Grayscale calibration for ^10^B quantification. Experimental calibration points (blue markers, with associated uncertainties) relating the image grayscale value to the corresponding ^10^B concentration are shown together with the best-fit calibration curve (red line). The resulting model was used to convert grayscale images into quantitative ^10^B concentration maps (ppm) for all subsequent analyses.

## Abbreviations

The following abbreviations are used in this manuscript:

GBM	Glioblastoma
RT	Radiotherapy
IDH	Isocitrate dehydrogenase
TMZ	Temozolomide
RBE	Relative biological effectiveness
PBCT	Proton Boron Capture Therapy
LET	Linear energy transfer
ROI	Regions of interest
