# Supplementary material for: Evaluation of a Boron-Conjugated SRC Inhibitor Combined with Proton and X-Ray Irradiation in U-87 MG and U-87 MG IDH1R132H Glioma Cell Lines
Source: Pharmaceuticals (Basel). 2026 Feb 28;19(3):392. doi: 10.3390/ph19030392 (PMC13028696; doi:10.3390/ph19030392)
Supplement: Supplementary file 1 [file pharmaceuticals-19-00392-s001.zip › pharmaceuticals-4126813-supplementary.pdf]

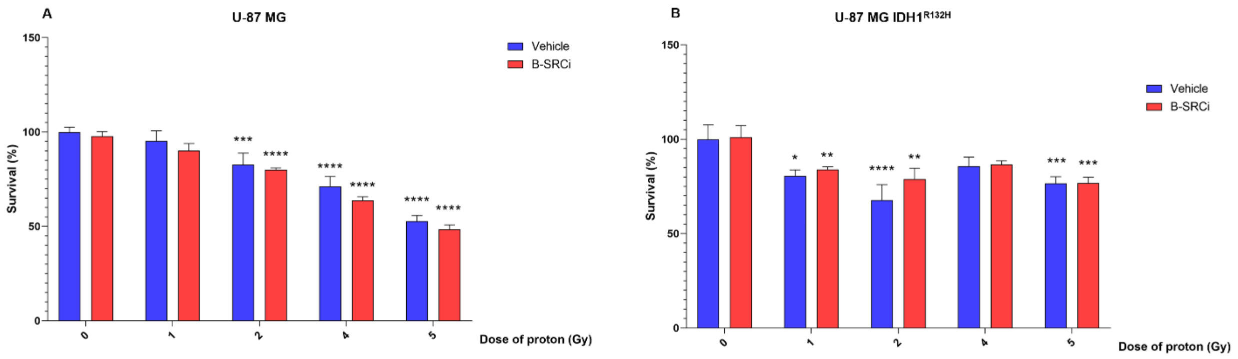

Figure S1. Evaluation of cell survival in U-87 MG (**A**) and in U-87 MG IDH1^{R132H} (**B**) cell lines irradiated with proton at 1, 2, 4 and 5 Gy after exposed to 10 µM of B-SRCi treatment; data were shown via interleaved bars, mean ± SD of n = 3 independent experiments; *p-value < 0.05; **p-value < 0.01; *** p-value < 0.001, **** p-value < 0.0001 vs 0 Gy vehicle (sham irradiated).

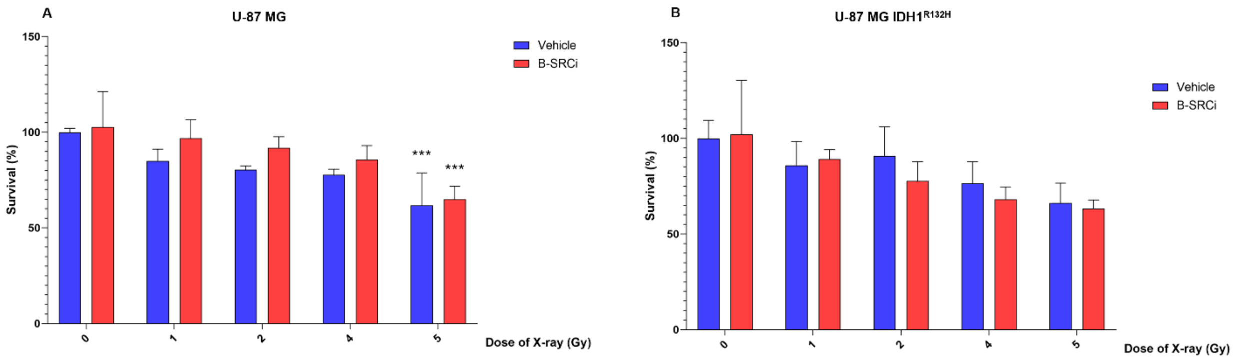

Figure S2. Evaluation of cell survival in U-87 MG (**A**) and in U-87 MG IDH1^{R132H} (**B**) cell lines irradiated with X-ray at 1, 2, 4 and 5 Gy after exposed to 10 µM of B-SRCi treatment; data were shown via interleaved bars, mean ± SD of n = 3 independent experiments; *** p-value < 0.001, vs 0 Gy vehicle (sham irradiated***)***.

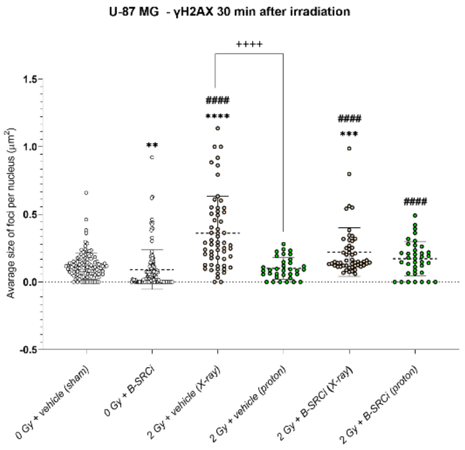

Figure S3. Average size of γH2A.X 30 min after proton and X-ray irradiation of U87-MG in combination B-SRCi.; data were shown scatter plot, mean ± SD of n = 3 independent experiments; **** p-value < 0.0001, *** p-value < 0.001 and ** p-value < 0.01 , vs 0 Gy vehicle (sham irradiated); #### p-value < 0.0001 vs B-SRCi; +++ p-value < 0.001.

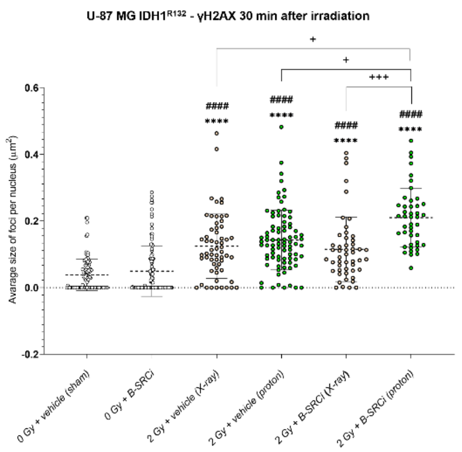

Figure S4. Average size of γH2A.X 30 minutes after proton and X-ray irradiation of U-87 MG IDH1^{R132H} in combination B-SRCi; data were shown scatter plot, mean ± SD of n = 3 independent experiments; **** *p-value* < 0.0001 vs 0 Gy vehicle (sham irradiated); #### *p-value* < 0.0001 vs B-SRCi; + *p-value* < 0.05 and +++ *p-value* < 0.001

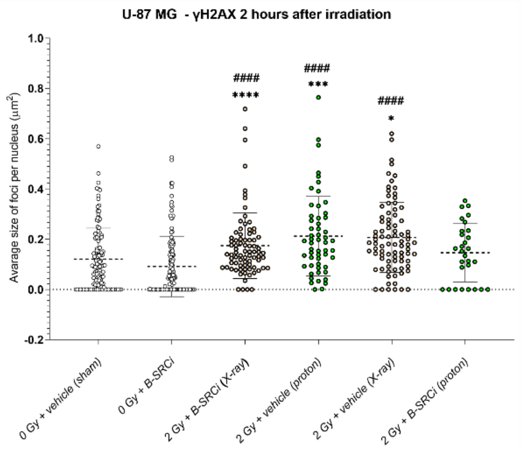

Figure S5. Average size of γH2A.X 2 hours after proton and X-ray irradiation of U-87 MG in combination B-SRCi.; data were shown scatter plot, mean ± SD of n = 3 independent experiments**p-value* < 0.05, ****p-value* < 0.001 **** *p-value* < 0.0001 vs 0 Gy vehicle (sham irradiated); #### *p-value* < 0.0001 vs B-SRCi.

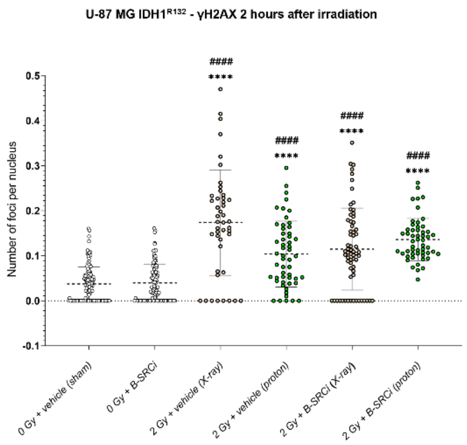

Figure S6. Average size of γH2A.X 2 h after X-ray and proton irradiation of U-87 MG IDH1^{R132H} in combination B-SRCi; data were shown scatter plot, mean ± SD of n = 3 independent experiments; **** *p-value* < 0.0001 vs 0 Gy vehicle (sham irradiated); #### *p-value* < 0.0001 vs B-SRCi.

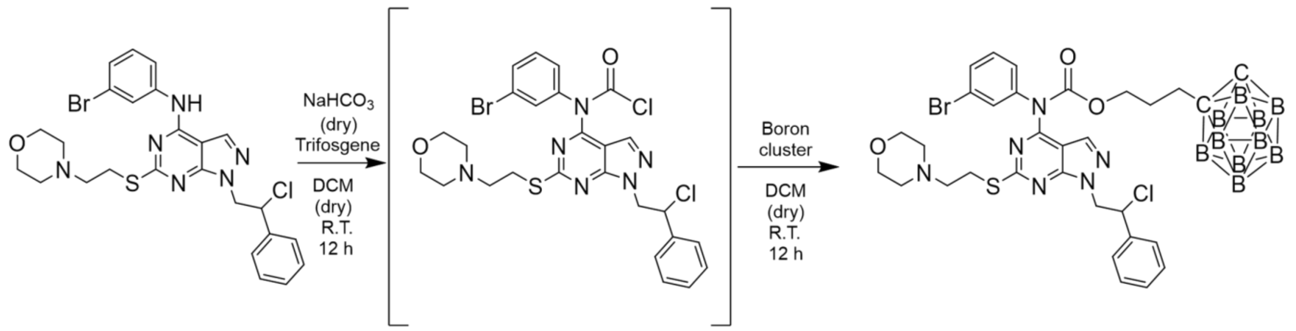

SI306 B-SRCi

Figure S7. Synthesis of the alcohol linker functionalized with o-carborane. Protection of the alcohol function of 3-bromo-1-propanol with DHP (**A**) is followed by nucleophilic attack of the boron cluster and displacement of the bromide atom (**B**). Finally, the functionalized linker is obtained by removing the protective group, restoring the hydroxyl moiety (**C**).

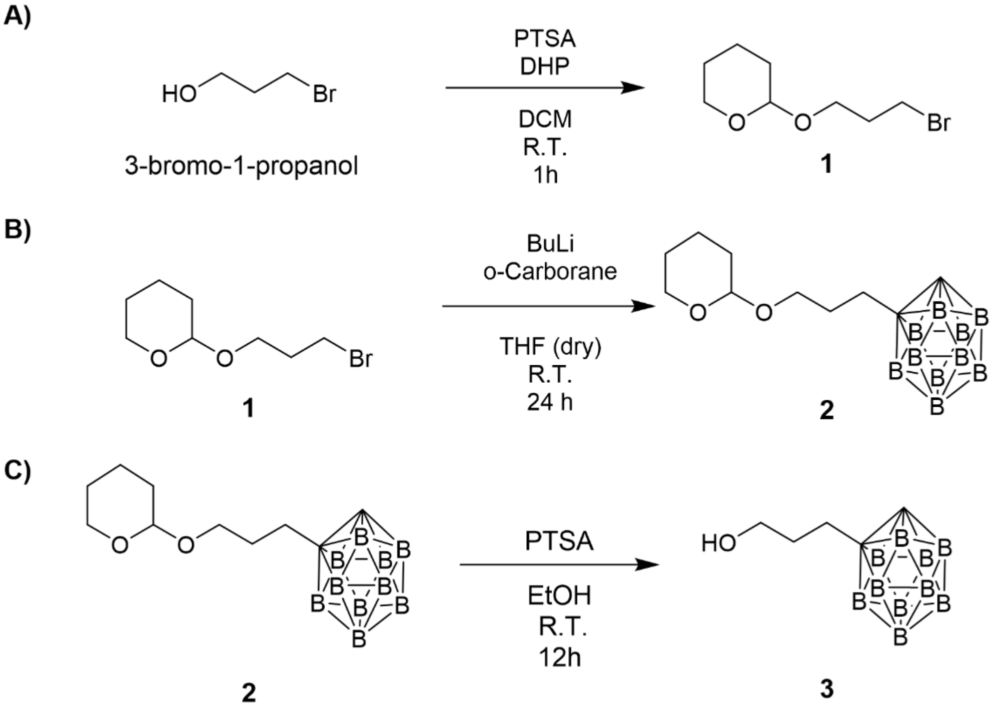

Figure S8. Detailed representation of the synthesis of an SI306 derivative hybridized with a boron cluster via the triphosgene route to give B-SRCi**.**

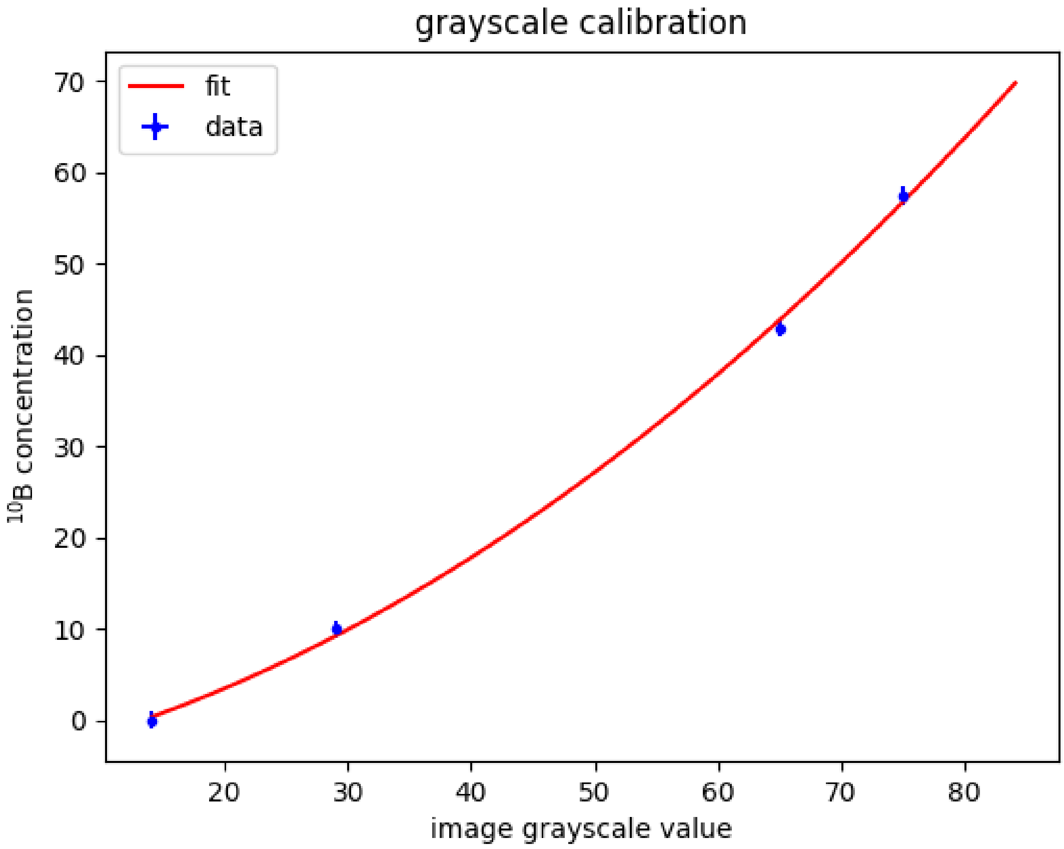

Figure S9. Grayscale calibration for ^{10}B quantification. Experimental calibration points (blue markers, with associated uncertainties) relating the image grayscale value to the corresponding ^{10}B concentration are shown together with the best-fit calibration curve (red line). The resulting model was used to convert grayscale images into quantitative ^{10}B concentration maps (ppm) for all subsequent analyses.
